# Guiding of keV ions between two insulating parallel plates

**DOI:** 10.1038/s41598-022-07905-x

**Published:** 2022-03-07

**Authors:** R. D. DuBois, K. Tőkési, E. Giglio

**Affiliations:** 1 Missouri University of Science and Technology, Rolla, MO 65409 USA; 2 Institute for Nuclear Research (ATOMKI), Debrecen, Hungary; 3 Centre de Recherche sur les Ions, les Matériaux et la Photonique (CIMAP), Normandie Univ, ENSICAEN, UNICAEN, CEA, CNRS, 14000 Caen, France

**Keywords:** Materials science, Physics

## Abstract

Experimental data are presented for low-energy singly charged ion transport between two insulating parallel plates. Using a beam intensity of approximately 20 pA, measurements of the incoming and transmitted beams provide quantitative temporal information about the charge deposited on the plates and the guiding probability. Using a smaller beam intensity (~ 1 pA) plate charging and discharging properties were studied as a function of time. These data imply that both the charge deposition and decay along the surface and through the bulk need to be modeled as acting independently. A further reduction of beam intensity to ~ 25 fA allowed temporal imaging studies of the positions and intensities of the guided beam plus two bypass beams to be performed. SIMION software was used to simulate trajectories of the guided and bypass beams, to provide information about the amount and location of deposited charge and, as a function of charge patch voltage, the probability of beam guiding and how much the bypass beams are deflected plus to provide information about the electric fields. An equivalent electric circuit model of the parallel plates, used to associate the deposited charge with the patch voltage implies that the deposited charge is distributed primarily on the inner surface of the plates, transverse to the beam direction, rather than being distributed throughout the entire plate.

## Introduction

Guiding of charged particles through insulating capillaries, i.e., transmission of charged particle beams resulting from a self-organized build-up of charge patches at various places on the capillary walls, was first investigated by Stolterfoht et al.^1^. That study showed that most of the transmitted ions kept their initial charge state, suggesting that the ions do not touch the inner wall of the capillary during the transport process. Based on this and numerous later studies, the accepted scenario is that some of the injected beam is guided though the capillary due to ions colliding with the inner surface of the capillary and depositing their charge. After some time, this accumulated charge generates an electric field strong enough to deflect a portion of the injected beam and prevent it from colliding with the surface. This process is self-supporting, i.e. a fraction of the injected beam will continue the charge-deposition to replace the charge loss due to the leakage currents, while the rest of the beam is guided through the capillary. A summary of these studies and their findings can be found in several review articles^2–4^. In general, however, the experimental studies have been qualitative in nature, e.g., typically an unknown portion of the beam is injected into the capillary and the guided beam intensity has been studied as a function of time after beam injection. Recently, however, Stolterfoht extracted absolute, rather than relative, transmission probabilities for some of the early data^5^. Such studies have been performed using both microscopic diameter capillaries in insulating foils like polyethylene-terephthalate (PET)^6,7^ or in solids such as silicon dioxide (SiO_2_)^8^ and aluminum oxide (Al_2_O_3_)^9,10^ and using macroscopic diameter glass capillaries^11–18^.

To provide additional information for testing theoretical models and simulations of the guiding process we previously reported quantitative data, e.g., absolute guiding efficiencies and measurements of the injected and guided currents as well as the current impacting the capillary walls, for low-energy argon ions injected into and transported though a macroscopic glass capillary^18^. For the geometry used, namely where the entrance surface and the outer cylinder surface were grounded, the charging and discharging data were shown to be compatible with a theoretical model^18,19^ where two discharge paths were identified. These paths accounted for charge flow away from the area where it was initially deposited, namely via (i) the bulk current, proportional to the bulk conductivity ${\kappa }_{b}$ of the glass capillary, and (ii) the surface currents, along the inner surface and proportional to the surface conductivity ${\kappa }_{s}$ of the glass-vacuum interface. Thus, for modeling the deposited charge decay, two decay rates, one for charge flowing along the insulator surface and one for charge migration through the bulk, were considered. For the cylindrical geometry used in^18^ the time dependence of the capillary charge was modeled by

1 $$Q\left(t\right)= {I}_{\text{dep}}\left\{f\frac{{\tau }_{\text{s}}}{a}\left(1-{e}^{-\frac{at}{{\tau }_{\text{s}}}}\right)+\left(1-f\right)\frac{\tau_\text{b}}{b}\left(1-{e}^{-\frac{bt}{{\tau }_{\text{b}}}}\right)\right\}+ Q\left(0\right){\{ fe}^{-\frac{a t}{{\tau }_{\text{s}}}}+ \left(1-f\right){e}^{-\frac{b t}{{\tau }_{\text{b}}}} \}$$

The current I_dep_ stands for the deposited charge per unit time at the inner cylindrical surface due to ion-surface collisions. The bulk decay time ${\tau }_{b}={\epsilon }_{r}{\epsilon }_{0}/{\kappa }_{b}$ is related to the bulk conductivity ${\kappa }_{b}$ and electric relative permittivity ${\epsilon }_{r}$ of the insulator, while ${\tau }_{s}={R}_{1} {\epsilon }_{0}/{\kappa }_{s}$ is related to the surface conductivities ${\kappa }_{s}$ and radius ${R}_{1}$ of the inner surface. The dimensionless constants $a$ and $b$ depend on the dimensions of the capillary and on the imposed boundary conditions for the electric field, typically grounded electrodes^19^. *f* is the fraction of the deposited charge that flows along the surface, and Q(0) is any residual charge left over from a previous beam injection. For brevity, we shall refer to the I_dep_ terms as the deposition portion and the Q(0) terms as the decay portion. Although the model leading to Eq. (1) is based on cylindrical geometry, the basic concepts should be applicable to the present geometry with the major difference being the constants a and b. As these are not known, the exponentials can be written using “effective” surface and bulk decay rates, e.g., exp(− t/τ_s eff_) and exp(− t/τ_b eff_).

However, a drawback of this study was that the injected beam intensity was not measured directly but had to be determined from an extensive analysis of accompanying current measurements. Although the final data of guiding as a function of the deduced deposited charge showed excellent agreement over a wide range of injected beam intensity, a more direct method is desirable. One such method was used by Giglio et al.^20^ where they used low-energy single charged argon ions to charge a macroscopic glass capillary until the transported beam intensity plateaued and then studied the decay of the capillary charge by moving the capillary such that the beam passed close to the outer surface. The deflection of this external beam was monitored as a function of time as the capillary charge decayed away. Although the measured deflection was directly proportional to the capillary voltage and hence the capillary charge, a limitation in the setup used was that the proportionality factor was unknown. A modified setup eventually lifted the limitation and the total charge accumulated in the tapered capillary could be measured^21^.

To go further in understanding the interactions between charged particles and insulating surfaces, the present experiment was designed. By using a simple Cartesian geometry of two parallel glass plates and an injected beam diameter smaller than the plate separation, the amount of beam entering the capillary could be measured, in contrast to most, if not all, of the previous studies where the beam diameter was larger than the entrance aperture. An aperture array allowed simultaneous injection of a guided beam between the glass plates plus two “bypass” beams traveling between the exterior side of one of the glass plates and a grounded metal plane could be simultaneously injected. Thus, simultaneous sampling of the internal and external fields produced by the deposited charge was possible. Finally, the parallel plate geometry allowed the apparatus to be easily modeled using SIMION software and approximated by a capacitor circuit.

Downstream from the guiding assembly, a position sensitive detector was used either in an unbiased mode for current measurements or in a biased mode for imaging the intensities and positions of the guided and external beams as a function of time after injection. The overall intent of this design was to associate the buildup of plate charge with the guided beam intensity and/or with the deflections of the external beams and to use SIMION simulations and an equivalent electric circuit model to connect the deposited charge with the plate voltage.

## Experimental method

**Figure 1:**
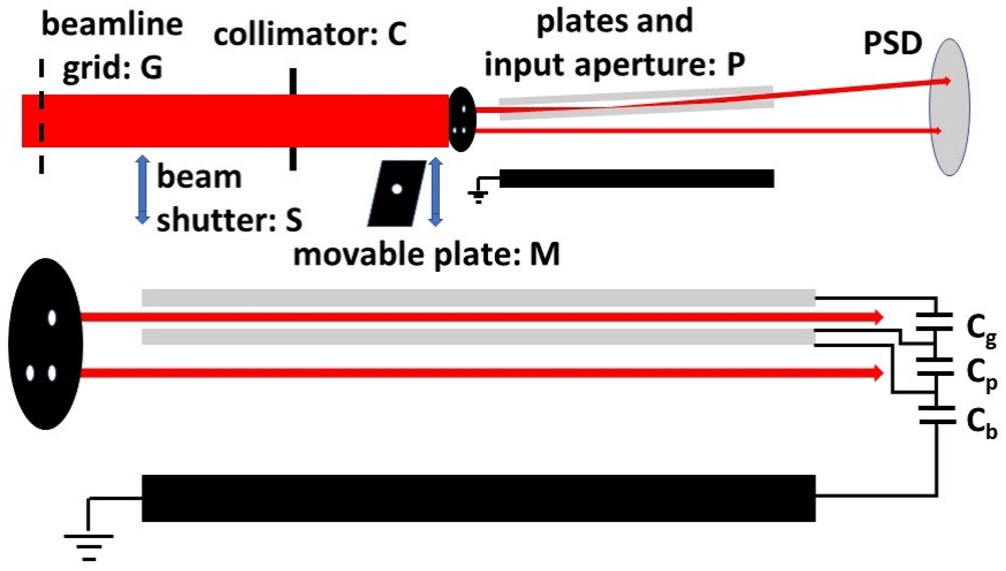
Upper portion: Overall schematic of the guiding assembly. Showing the guided and bypass beams in a tilted plate configuration. Lower portion: an expanded view showing the plate assembly aligned with the beam axis. The length is not to scale and the three capacitors illustrate the equivalent electric circuit model used and are NOT part of the experimental setup.

The experiment was performed at the Missouri University of Science and Technology using a 1 keV Ar^+^ beam produced by a differentially pumped commercial ion sputter gun and, similar to a study performed by Pokhil et al.^22^, a guiding assembly consisting of two parallel glass plates (Fisher Scientific Co. type 12-549 microscope slides) 58 mm long, 25.4 mm wide and 0.925 mm thick. The plate separation was 1.25 mm. The plates were set in parallel shallow grooves in an aluminum holder, which was connected to an ammeter. Thus, the bottom borders of both glass plates were electrically grounded while all other surfaces were insulators. Grooves in a Vespel plate at the entrance end kept the plates parallel in the vertical direction. Attached to the aluminum holder was a 50 mm long grounded plane positioned an average of 7.5 mm away (7.3 and 7.8 at the entrance and exit ends) from one of the glass plates. Mounted on the Vespel plate was an aperture array consisting of three 0.5 mm diameter apertures. (See Fig. 1). One aperture, centered between the plates, produced a “guided beam”. The other two, offset horizontally by 3 mm and vertically by 0 and 1.75 mm with respect to the guided beam, were used to produce “bypass beams”. The aperture and plate assembly, P, were mounted on a rotatable xyz manipulator.

The upper portion of Fig. 1 shows an overall schematic of the setup. A 6 mm diameter collimator, C, approximately 2 cm upstream from the input aperture array and a defocused beam were used in order to simultaneously illuminate all three input apertures as uniformly as possible. Based upon the ion source lens position and geometrical factors the total beam divergence for the guided and bypass beams was estimated to ~ 0.1° and the plate rotation angles are estimated to be accurate to ± 0.05°. A metal plate on a horizontal manipulator, M, located between the collimator and the three input apertures could be positioned to block the guided beam without disturbing the bypass beams or, using a small hole in this plate, to block the bypass beams and allow just the guided beam to pass.

To monitor the guided and bypass beams, a 50 mm diameter channelplate with a 2D anode, PSD, was located approximately 16 cm downstream from the center of the glass plates. With only a small bias voltage applied, the transmitted currents impacting the channelplate could be measured using an electrometer or when the beam intensity was reduced and high voltage was applied, images and intensities for each of the guided and bypass beams could be recorded. To ensure stable conditions, the beam current was monitored using either a grid in the beamline, G, the current on the input aperture array, or the current on the movable plate attached to the horizontal positioner. In addition, the current impacting and ultimately exiting the glass plates through their lower edges into the aluminum holder was recorded. Outputs from the electrometers were digitized and sent to a PC with a second PC used for the imaging measurements.

This parallel plate setup, provided a well-defined geometry for the initial charge patch production and predictable electric field conditions for both the guided and the bypass beams. It also allowed using an equivalent electric circuit model with capacitors where the only unknown parameter was the plate area. Figure 1 shows the circuit where the capacitors are between the plates, C_g_, across the bottom plate, C_p_, and across the bypass region, C_b_.

## Results

### Simion trajectory, electric field simulations and equivalent electric circuit model

To aid in interpreting the data, a post-experiment SIMION model of the apparatus and simulations of the guided and bypass beam trajectories was used. In the model, a voltage profile appropriate for a uniform circular beam impacting on a tilted plate was applied at the inner surface of the lower plate within the geometrically accessible region of the initial charge patch with the potentials for the rest of that plate and for the other plate set at 0 V. A known number of ions were injected at either 2° or 3° through the guiding and bypass apertures and information about the number and locations of where ions impacted each plate, the number of ions exiting between the plates, and the position of the bypass ions at the location of the 2D detector were recorded. This was done for many voltages between 0 and 40 V and a few voltages between 300 and 1000 V.

**Figure 2:**
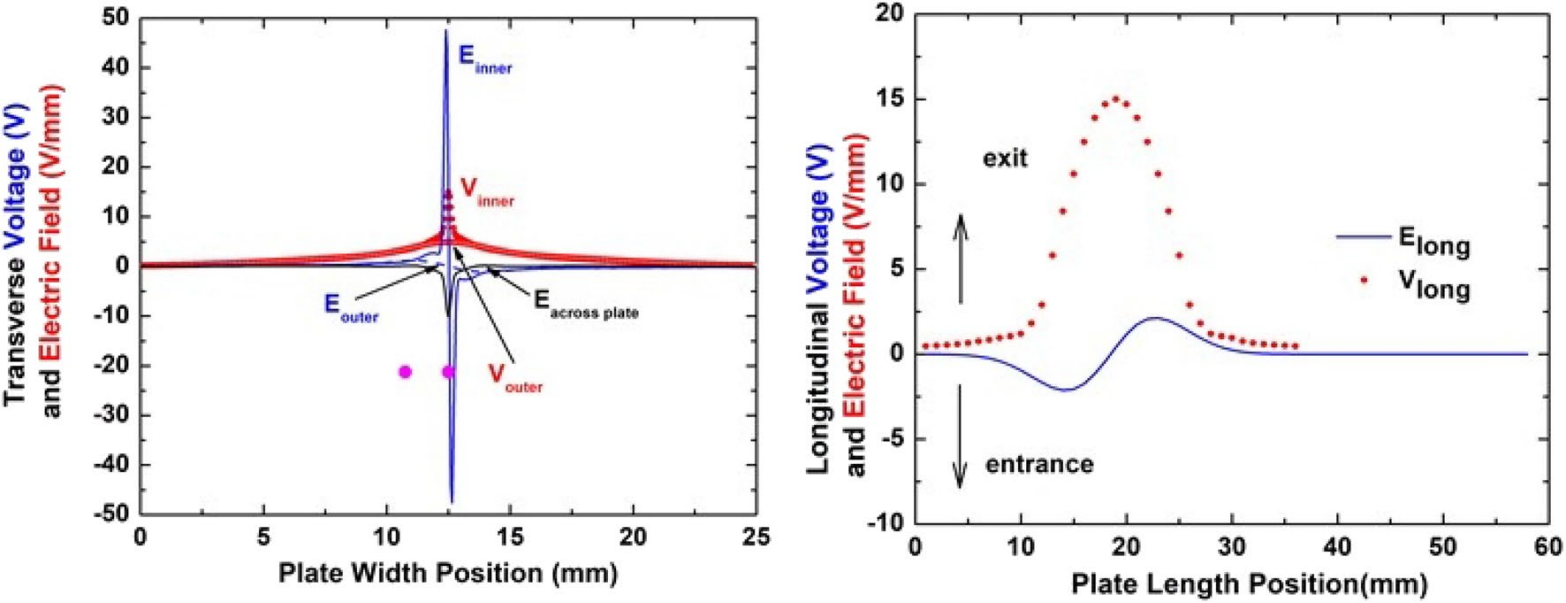
SIMION predicted voltages (red symbols) and electric fields (blue lines) transverse and along the beam direction due to a charge patch on the lower glass plate in the upper portion of Fig. 1. Filled symbols, indicated by V_inner_, and solid lines are along the inner surface; open, indicated by V_outer_, and dashed lines are along the outer surface; the black curve is the internal field of the glass plate pointing outwards from the inner to outer surface. For the transverse (longitudinal) fields, + , − indicates field direction toward the 25, 0 mm side (toward the exit, entrance ends). All examples are for a 15 V maximum potential of the patch voltage. The solid magenta circles illustrate the magnitude of the fields at the location of the two bypass beams.

In addition to the trajectory information, our SIMION model provided information about the voltage and electric field profiles transverse to and along the beam direction. The symbols in Fig. 2 show the voltage profiles for the situation where the maximum patch voltage is 15 V. The transverse profiles imply that the charge patch has an effective width, i.e. the region containing most of the integrated intensity, *w*, of 3.4 and 9.9 mm along the inner and outer surfaces. In the longitudinal direction, the effective length, *l*, is 60% of the geometric length, i.e., 60% of 10 and 16 mm for our plate rotations of 3° and 2°.

Using these voltage profiles, the electric fields transverse to the center of the charge patch and along the beam direction were determined (the solid lines in Fig. 2). In the transverse direction (the left figure) the lower and upper edges of the plates are at 0 and 25 mm and in the longitudinal direction (the right figure) the entrance and exit ends are at 0 and 58 mm. In the transverse direction, +,− values indicate the directions of the electric fields along the surfaces (inner surface: solid lines; outer surface: dashed lines) are away and toward the grounded aluminum holder with the negative value also indicating the field within the bulk is pointing from the inner to the outer surface (the black curve). In the longitudinal direction, +, − values indicate the field along the inner surface is directed toward the exit and entrance ends respectively.

As seen, the transverse field along the inner surface is very strong near the physical patch dimension with a much weaker component extending out to ~ 5 mm. The next strongest field, also quite localized, is directed through the plate toward the outer surface. For comparison, the fields on the inner surface along the beam direction and in the transverse direction along the outer surface are significantly weaker. These fields thus indicate that the primary charge flow, i.e., decay mode, is along the inner plate surface in the transverse direction from where it was deposited. Integrals of the electric fields imply that this surface decay mode is approximately 12 times more probable than the bulk decay mode, i.e., *f* in Eq. (1) is ~ 0.92. It should be noted that because only one edge of the plate is in contact with ground, the portion of charge driven in the opposite direction will weaken the positive transverse field as a function of time. As a final comment, the magenta symbols indicate the relative transverse positions of the bypass beams. Note that for the situation shown where the charge is located at the original deposition area, the outer plate voltage is similar for both bypass beams.

To associate the SIMION patch voltage information with the deposited charge information from our current and imaging studies, our parallel plate configuration was modeled as an equivalent electrical circuit of capacitors, as was illustrated in Fig. 1. The simple model used assumes that the inner surface of the lower plate is connected to ground via a parallel combination of capacitors, one between the inner surfaces of the two plates, C_g_, and a series combination of capacitors between the inner and outer surfaces of the lower plate and between the outer surface of the lower plate and the grounded plate, C_p_ and C_b_. In units of ε_o_A, where A is the area, C_g_, C_p_, and C_b_ have values of 0.8, 4.8 and 0.13. We note that our SIMION study showed that until maximum transmission is achieved, no beam strikes the upper plate; therefor our model assumes the upper plate to be at ground potential. This model and Eq. (1) provide a connection between the temporal deposited charge information from the experimental studies and the SIMION voltage information with the unknown parameter being the capacitor plate area. Because of the mapping of the circular beam on the tilted plate, the minimum area would be an effective patch dimension, 0.6* l* × 3.4, at maximum the entire plate area, 58 × 25, with a more probable area being the effective patch length times the plate width, 0.6* l* × 25.

### High current study

To provide quantitative information associating beam guiding with plate charging and discharging properties, a beam current of ~ 20 pA and a plate rotation of 3° was used. Thus, the guided beam impacted the primary plate and deposited charge within a region between 7 to 17 mm from the entrance end (see Fig. 1) with an intensity distribution corresponding to mapping the input aperture on the tilted plate. For this study, the bypass beams were blocked and the time dependences of the current exiting the lower edge of the glass plates, I_plates_, and the transmitted (guided) current, I_trans_, were measured. To ensure that the beam intensity did not change with time, the beam current sampled on the collimating aperture was also monitored, I_coll_.

We note that the plate current, I_plates_, is the sum of the leakage current, i.e. the deposited charge leaking to the grounded bottom edge of the plates due to the bulk and surface conductivity of the glass plates, and, in principle, the displacement current which accounts for the appearance of negative surface charges at the metal holder because of positive charge being added to the glass plates. As the accumulated charge changes with time, the amount of negative surface charges varies too, resulting in electric currents that are monitored by the ammeter. However, in our setup, the displacement current is expected to be small so that I_plates_ primarily provides information about the charging rate of the glass plates by the injected beam. Also, in principle, included in the I_plates_ current is any loss of charge associated with secondary electrons. However, a + 10 V bias applied to the aperture array suppressed any possible contamination at the entrance and considering the geometry, loss of secondary electrons produced at the charge patch area is extremely unlikely. Also, ionization of the residual gas was insignificant.

**Figure 3:**
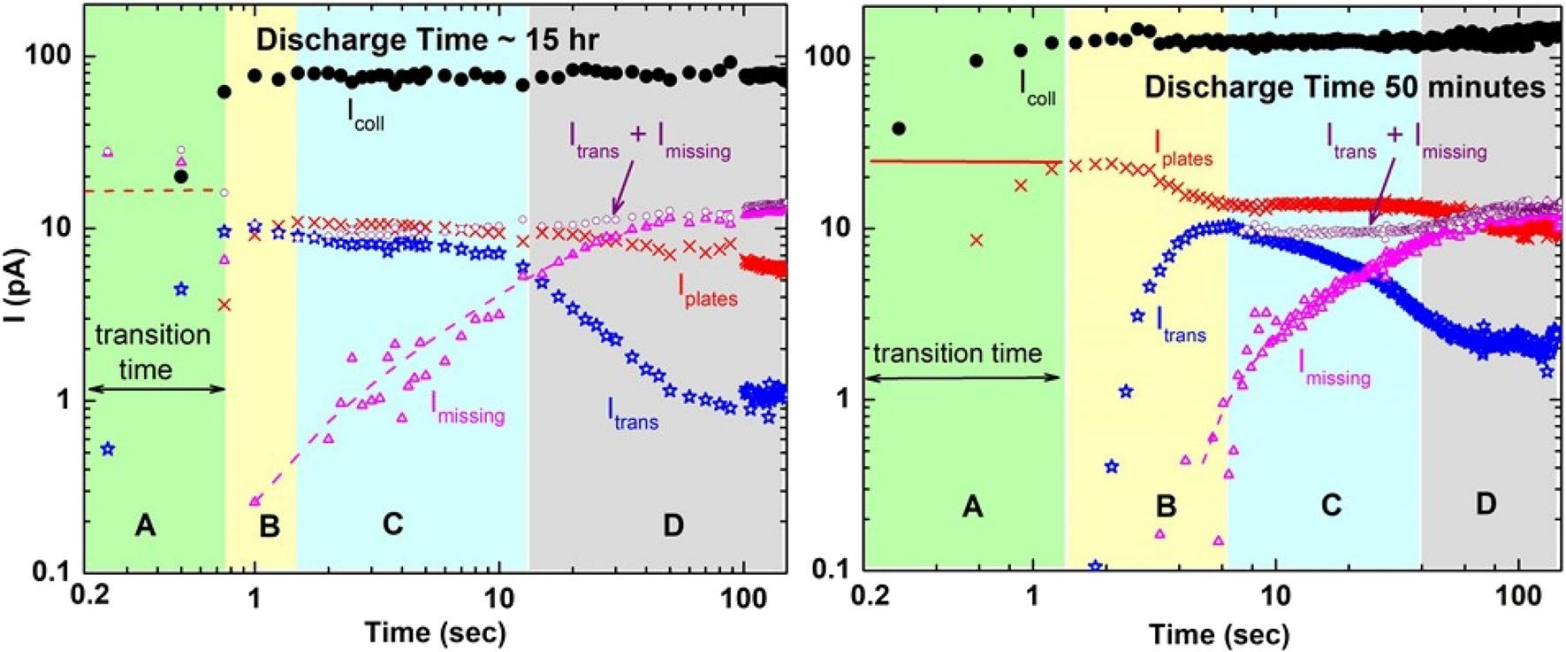
Currents measured at the input aperture array, I_coll_, (filled black circles), at the aluminum holder, I_plates_, (red x’s), and transmitted through the guiding assembly, I_trans_, (open blue stars), following a 15 h and a 50 min discharge time. In the right figure, the solid red line is an eyeball extension of the maximum value of I_plates_. In the left figure, the dashed red line is a similar extension with magnitude based on the 50 and 60 min discharge data. The open magenta triangles and dashed curves show an unaccounted for current, I_missing_, obtained via a subtraction process as described in the text. The open purple circles show I_trans_ + I_missing_. The colored regions A-D indicate regions associated with various guiding processes. See text for details.

Figure 3 shows these data for two situations where the plates were allowed to discharge for 15 h and for 50 min. An additional set of data following a 60 min discharge time was also measured but is not shown as the same qualitative features are seen. Quantitatively, the onset for guiding for the three data sets is slightly shifted in time. This could be associated with an interplay between the very rapid charge deposition and the time during which the valve was opening rather than indicative of any residual plate charge. But, as will be discussed later, these shifts are interpreted to differences in the deposited charge distributions which lead to different guiding fields.

The sampling rate for the 50 min discharge time data was ¼ second; for the 15 h discharge time, the sampling rate was ¼ second for the first hundred seconds after which it was changed to 1 s. In addition, there was approximately a 55–60 ms processing time for each data point. There are four distinct regions, A–D, clearly seen in our 50 and 60 min discharge data whereas for the 15 h discharge data, regions A and B fall within the transition time window; hence, are hidden. Therefore, the general features after the valve used to block the beam is opened are: Initially, region A, there is a transition time of 0.5–1 s where the measured currents are apparently influenced by either the electrometer response times or by the time required to open a valve. Around the border between regions A and B, I_plates_, the red x’s, remains nearly constant for a brief time. The horizontal red lines are extensions of this through the transition time region.

In region B, I_plates_ decreases by roughly a factor of 2 and the guided current, I_trans_, shown by the blue open stars, increases from a very small value or zero to ~ 10 pA, implying that the maximum guiding efficiency for this parallel plate setup is approximately 50%. For a 3° plate rotation, our SIMION modeling revealed that in region A after the primary plate patch voltage has increased to a certain value, some of the injected beam is slightly deflected and impacts downstream from the geometric region. With increasing voltage, this continues until as much as 30–40% of the incoming beam intensity is distributed along the downstream plate region. The modeling also implies that the decrease in I_plates_ and increase in I_trans_ in region B occurs when the patch voltages cause more and more beam to be reflected and exit between the plates thus reducing the number striking the first plate.

In region C, I_plates_ remains constant while I_trans_ decreases to some tens of percent. Finally, in region D a slow, systematic decrease in I_plates_ and a stronger decrease in I_trans_ to roughly 10–20% of its maximum value are seen. Two other curves, I_missing_ (the open magenta triangles and dashed line) and I_trans_ + I_missing_ (the open purple circles) are also indicated in these regions. These were obtained from the measured I_plates_ and I_trans_ currents in the following manner. As previously noted, the beam current on the collimating aperture, I_coll_, is constant. Thus, the sum of I_plates_ and I_trans_ should also be constant. However, the sum was found to slowly decrease, implying a portion of the injected beam is not accounted for. We refer to this as I_missing_ and determined it by subtracting I_plates_ + I_trans_ from the sum of these currents immediately after the beam is injected, the horizontal red lines. Although a previous guiding study^20^ showed that stray electrons can be important and stray electrons could be the source of I_missing_, the bias on the collimating aperture plus geometry tends to rule out any upstream stray electron contamination and geometry plus the electric field from a downstream deflection system used to center the guided beam on the channelplate tends to rule out any downstream contamination. Therefore, because I_missing_ + I_trans_ is found to have a constant magnitude matching the maximum I_trans_ value throughout the entire region C, we attribute I_missing_ to be a portion of the guided current being lost due to focusing/partial blocking caused by the combination of voltages of charge patches on the the lower and upper plates. If this lost portion ends up on the collimator and/or the Vespel plate on which the collimator is attached or exits the plate region and misses the downstream detector, it would not be measured. Unfortunately, although the addition of the two currents strongly implies this scenario, we cannot definitively confirm it. However, note that in region D the sum of I_missing_ + I_trans_ increases in the same region where strong blocking is observed. This, plus our SIMION modeling which showed strong focusing and ever increasing blocking of the beam for primary and secondary patch voltages several hundred volts and higher, supports the above scenario.

### Charging, discharging study

Using lower beam currents, ~ 0.85 pA, the guided beam intensity was studied. Again, the bypass beams were blocked and the plate rotation was 3°. Because the beam intensity was at the minimum of the electrometer sensitivity, I_plates_ has a rather large uncertainty or ~ 30%. The guided beam was measured using the channelplate detector in pulse counting mode. As this injected current is approximately 25 times smaller than that used for the Fig. 3 data, the times required for onset of guiding and to reach saturation are proportionally longer and leakage currents limit the maximum guiding probability to approximately 3.5%. Thus, the charge deposition portion of Eq. (1) can be used as written.

For this study, the beam was admitted long enough for the guided beam intensity to saturate, meaning that for this beam intensity the plates were charged to their maximum value. Then, the plates were allowed to partially discharge by blocking the injected beam for times ranging from a few seconds to many tens of minutes. After the blocking was removed, the transmitted intensity was measured until maximum transmission was again achieved. The process was repeated for a different blocking, i.e., discharge, time. To ensure that the beam intensity remained constant, the currents at the beamline grid and the collimating aperture were measured. The exception to this procedure was the first measurement after the beam being blocked overnight. In this case, a brief pre-decay injection, ~ 1 min was used prior to the blocking time of 60 min. Thus, only a negligible amount of charge was deposited meaning that for this set of data the plates are considered to be totally discharged.

**Figure 4:**
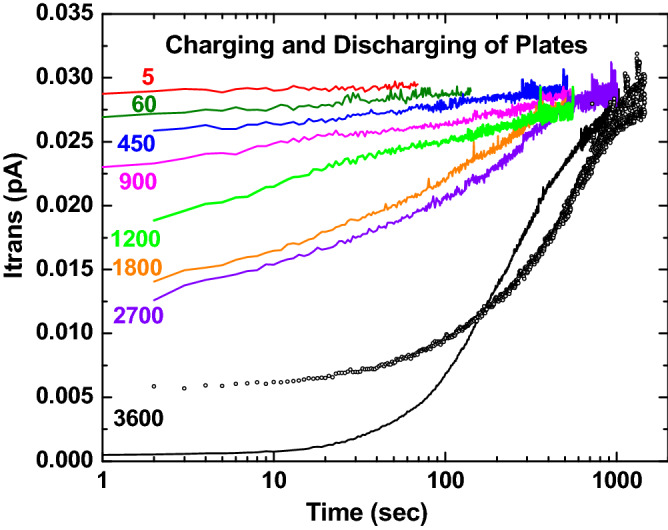
Transmitted beam intensity as a function of time following various plate discharge times in seconds. The solid black curve shows the initial measurement in the sequential series with the open black circles showing a repeat measurement on the following day.

Figure 4 shows the temporal dependences of some of these data where it is seen that some of the curves have not reached a full saturation value when the run was terminated. The solid curves are for data collected sequentially for discharge times beginning with a 60 min discharge time, followed by times increasing from 5 s to 45 min whereas the open circles show a 60 min discharge time measured the following day.

**Figure 5:**
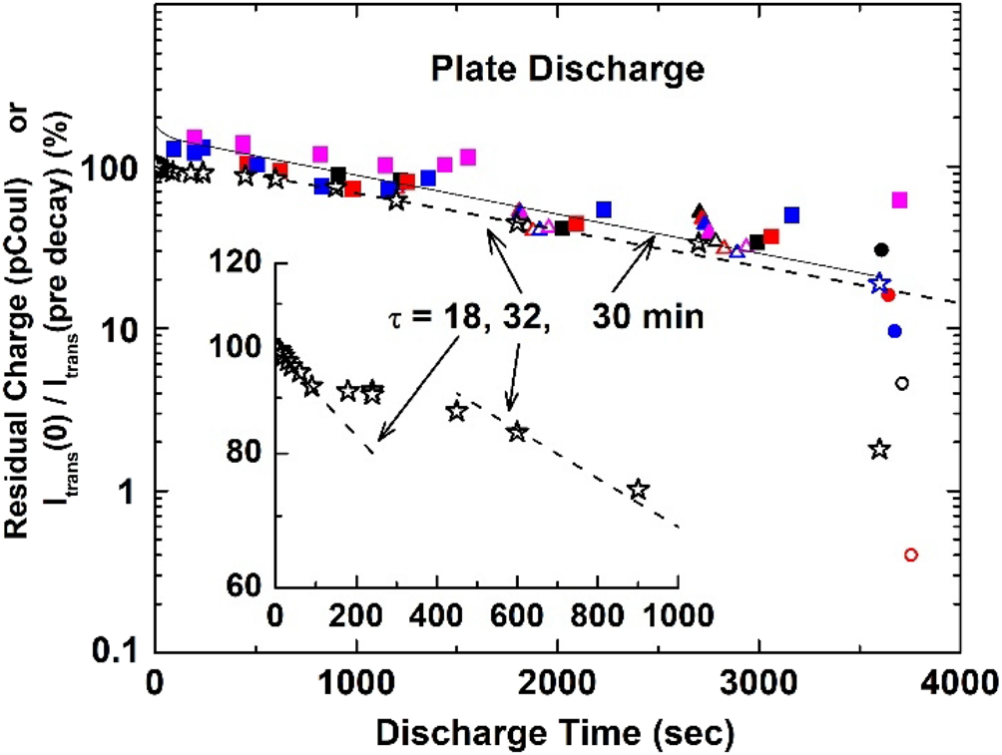
Open stars and dashed lines: Ratio of transmitted beam intensity, in percent, just after various blocking times with respect to the intensity just before blocking. The sequentially measured data are shown in black and the repeat measurement for a 60 min decay time is shown in blue. The inset is an expanded view of these data. Filled and open squares, triangles and circles and solid line: Residual plate charge, in pCoul, for various transmitted beam intensities obtained as described in the text. The lines are exponentials with time constants τ.

These data, which show a systematic decrease in the initial guided intensity with increasing discharge time due to some remaining residual charge deposited in a previous run, were analyzed in two ways. One was based on a method first used by Stolterfoht et al.^1^ Here, the ratio of transmitted beam intensity measured immediately after beam blocking to the intensity prior to blocking the beam was used with the results shown by the open stars in Fig. 5. The black stars are for data measured sequentially in one day with the repeat measurement on the following data after a 60 min discharge time shown by the blue open star. Although the overall behavior of these data is compatible with a single exponential decay, the expanded view in the inset indicates an initial exponential charge decay rate, followed by a plateau, after which a second, slower exponential decay takes place. Dashed lines and time constants for these exponential regions are given. The plateau region separating these regions is in conflict with accepted models which assume two charge decay modes, one associated with charge flow along the insulator’s surface and one through the bulk. Also, attempts to use these decay times in Eq. (1) to compress the temporal data in Fig. 4 into a single guiding probability as a function of charge were unsuccessful.

Therefore, a second analysis method that we used in a previous study of guiding through an insulating cylinder^18^ was used. For this method, the charge deposition for each curve shown in Fig. 4 was calculated using the I_dep_ portion of Eq. (1), i.e., neglecting the Q(0) term. Then, for a fixed value of the transmitted beam intensity, the amount of residual charge required to bring each set of data into agreement was found by subtracting the calculated deposited charge for each set of data from the value when the plate was totally discharged. This was done for many transmitted beam intensities using an iterative process where the parameter *f* and effective decay times were adjusted in order to compress the curves. Again, no combination could be found that worked, e.g., parameters required to shift the curves to the right in Fig. 4, in order to match the 3600 decay time data, always yielded poor agreement for the shorter times after beam injection. During this process, it was noted that good overlap of all the curves resulted if it was assumed that both decay modes were active while charge was being deposited, i.e., the I_dep_ portion of Eq. (1), but only the bulk decay mode was important after charge deposition ceased, i.e., in the Q(0) portion of Eq. (1).

To understand this, beginning with a fully discharged system, when beam is injected at each time step, ∆t′, q(t′)) = I_dep_∆t′ charge is deposited and then begins to decay exponentially with time. In Eq. (1), the deposition portion is the sum of these assuming a) that a fraction of the the total deposited charge Q decays via surface currents with the remainder decaying via the bulk and b) that the incoming beam is constant and the residual charge portion assumes that at some earlier time the plates are instantly charged to some value which then begin to decay. However, if I_dep_ is time dependent, in a sequential series of measurements each having charge deposition for time ∆T_τ_ following a discharge time of d_i_ such as those presented here, for all but the initial measurement where the plates are totally discharged, the total residual charge at time t > t′ is

2 $$Q\left(t\right)=\sum_{{t}^{^{\prime}}} q(t{^{\prime}})\left\{f{e}^{-(t- {t}^{^{\prime}})/{\tau }_{s eff}}+\left(1-f\right){e}^{-(t- {t}^{^{\prime}})/{\tau }_{b eff}}\right\}$$

Here, τ_s eff_ and τ_b eff_ are the effective decay rates mentioned previously. Therefore, when I_dep_ is constant, for measurement *i*, Eq. (1) becomes

3 $$Q\left({t}_{i}\right)=\left\{{ q}_{\text{dep s}}\left(1-{e}^{-\frac{{t}_{i}}{{\tau }_{\text{s eff}}}} \right)+{ q}_{\text{dep b}}\left(1-{e}^{-\frac{{t}_{i}}{{\tau }_{\text{b eff}}}}\right)\right\}+\left\{{{q}_{res s}e}^{-\frac{{t}_{i}+{d}_{i}}{{\tau }_{s eff}}}+ {{q}_{res b}e}^{-\frac{{t}_{i}+{d}_{i}}{{\tau }_{b eff}}}\right\}$$

where the maximum values for the surface and bulk deposited and residual charges are q_dep s_ = I_dep_*f*τ_s eff_, q_dep b_ = I_dep_(1−*f*) τ_b eff_, ${q}_{res s}=f{\tau }_{s eff}\left(1-{e}^{-\frac{{\Delta T}_{i-1}}{{\tau }_{s eff}}}\right)$, ${q}_{res b}=(1-f){\tau }_{b eff}\left(1- {e}^{-\frac{{\Delta T}_{i-1}}{{\tau }_{s eff}}}\right)$.

**Figure 6:**
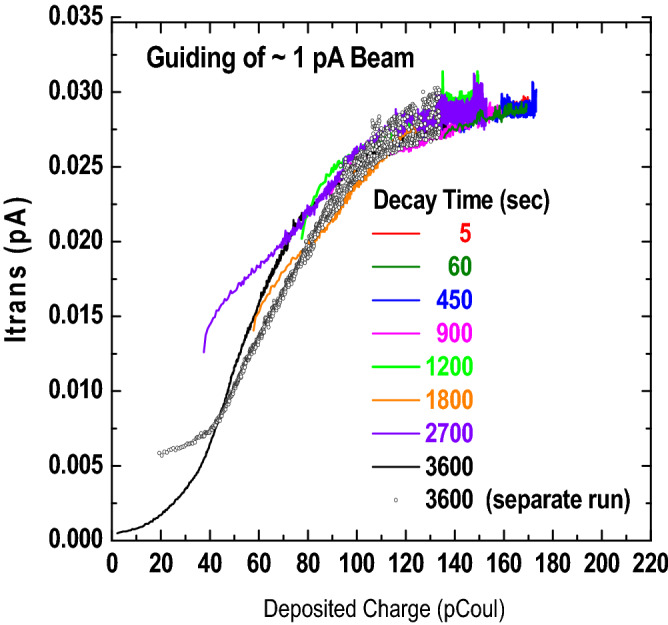
Transmitted beam intensity as a function of deposited charge determined as described in the text.

An important difference with respect to Eq. (1) is that depending on the respective values of τ_s eff_, τ_b eff_, d_i_, and the deposition times ∆T, the relative importance of the residual charge for the surface and bulk modes of decay changes. We also point out that Eq. (3) assumes only the previous measurement contributes to the residual charge. But, again depending on the decay rates and the deposition and discharge times, for a sequential set of measurements the residual charge from more than one previous measurement may be important. By using Eq. (3) with parameters *f* = 0.85, τ_s eff_ ~ 40–45 s and τ_b eff_ ~ 1800–2100s good, although not perfect, compression of the data is obtained, as seen in Fig. 6 and by the filled circles, squares and triangles in Fig. 5. From the discussion of Eq. (1), the relationship between the effective and “material” decay times is τ_s eff_ = τ_s_/a and τ_s eff_ = τ_b_/b where a and b depend on geometry, electric fields and boundary conditions. Comparing the present decay times with those we found using a cylindrical capillary with grounded outer surface implies that four our parallel plate geometry a and b are ~ 0.17, i.e., ~ 1/6. We also note that SIMION simulations of the patch to ground electric field, i.e., the radial field for a cylinder and the transverse field for our parallel plates, show that for our parallel plate configuration, the field is approximately 20 times weaker. This static picture will change in time due to charge migration but is consistent with the longer decay times we find.

### Imaging study

For the final study of this work, the beam intensity was reduced to ~ 25 fA which further increased the amount of time required to reach maximum transmission and permitted images of the intensities and positions of the guided and bypass beams to be obtained as a function of time. At this low beam intensity only the current on the grid in the beamline could be read by the electrometers. Therefore, the grid currents and the guided beam intensities at 25 fA, 0.85 pA and ~ 20 pA were used to estimate I_plates_ for this imaging study. Doing so gave an estimated beam intensity of 25 ± 20 fA.

Using this very low intensity, images of the guided and bypass beams were obtained by first blocking the guided beam was for an hour, then reinjecting it and collecting images for 1 min at various later times. Unfortunately, and unexpected, the post-experiment analysis of these images showed no significant, easily measurable, deflections. To provide greater sensitivity, projections of horizontal slices through the bypass beam spots in the images were used to generate bypass beam intensity profiles for various times after reinjection. These profiles were fitted with a Gaussian plus an exponential function added on the higher deflection side. The Gaussian magnitudes, widths, and centroids were adjusted to provide the best overall agreement and all fits were then scaled such that their integral intensity was the same. The same scaling was also applied to the data points. Finally, the various scaled data and fits were subtracted from the reference profile (shown in the upper left corner of the figure) which was measured long after beam injection. In some cases, small (< 10%) changes in the magnitude of the non-reference fits were made in order to yield better agreement. We note that although both bypass beams yielded approximately the same deflections, our analysis used the bottom bypass beam (the beam located at ~ 11 mm in Fig. 1) data which was slightly more intense.

**Figure 7:**
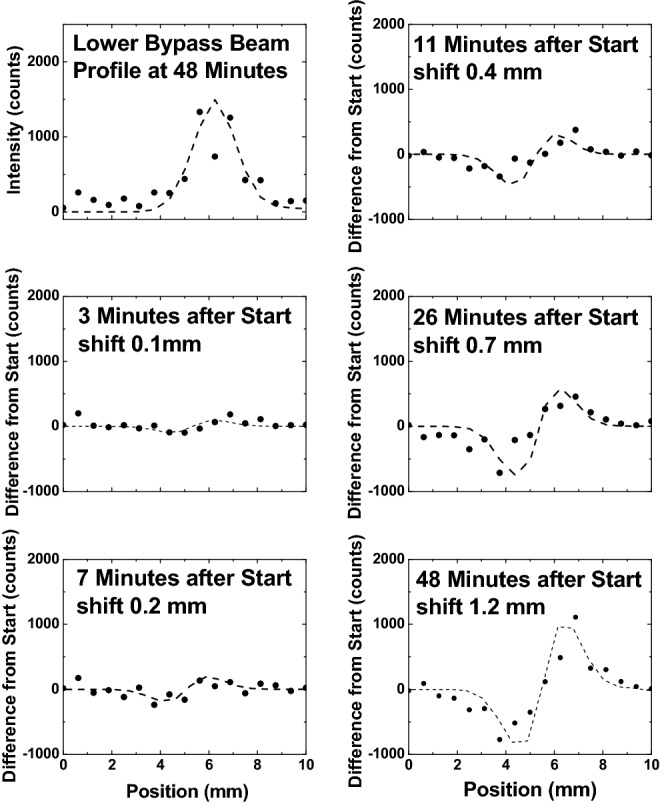
Left, top: measured, the solid dots, and fitted, the dashed line, horizontal profiles of the lower bypass beam. These were used as reference values. Other figures show differences between profiles measured and fitted at various times after beam injection with the reference values. The listed shift values are differences in the fitted peak positions used in each case. See text for details.

Measured values and fitted values determined in this manner are shown in Fig. 7 with the “shifts” between the various profiles and the reference profile that provided the best visual agreement listed in each case. To understand these figures, the reader is reminded that subtracting two Gaussian profiles that are offset from each other will yield a dip and peak. Small offset values produce a shallow dip and a small peak. As the shift becomes larger, the depth and height of these features increase. If the two Gaussians have different intensities, the dip and peak will have different magnitudes. As seen, this procedure produces a systematic deflection as a function of time, i.e., with deposited charge. We also found that the measured deflection was proportional to the intensity of the guided beam component.

### Combination of all studies

Using the method outlined above, the amount of charge deposited for our high current and imaging studies was determined. Then, these data were associated with our SIMION information using the equivalent electric circuit model to calculate the patch/plate voltages from the deposited charges. As stated previously, in this conversion the capacitor plate area is an unknown with, initially, the minimum area being the effective patch length times the effective width, i.e., 0.6* l* × 3.4 mm with *l* = 10, 16 mm for the 3°, 2° plate rotations and the minimum width taken from the inner surface voltage profile. At later times the probable maximum area is approximately the effective patch length times the plate width (0.6* l* × 25 mm^2^).

**Figure 8:**
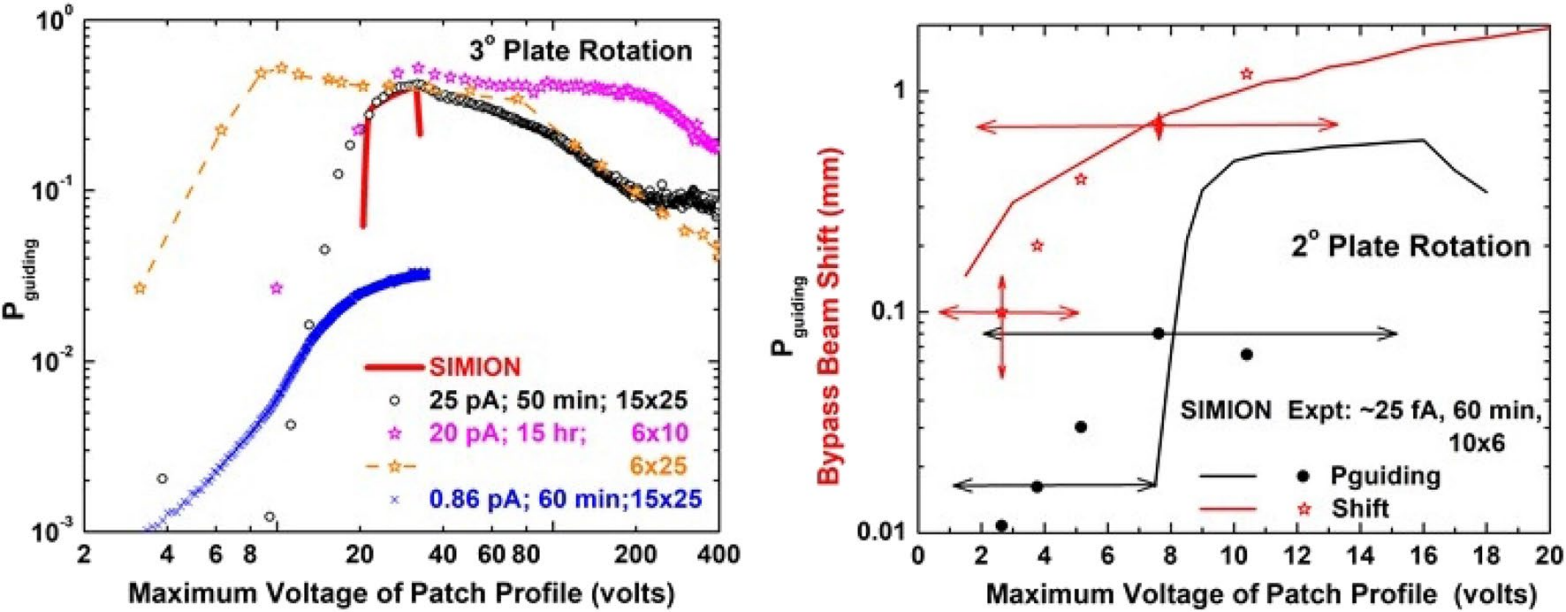
Guiding probabilities and bypass beam deflections as a function of the maximum voltage of the charge patch profile. Left figure is for a 3° plate rotation with the solid red line showing the SIMION predictions and the symbols showing the experimental data. The beam current, discharge time prior to data collection, and the estimated effective dimensions of the charge patch are listed for each dataset. The right figure is for a 2° rotation with guiding probabilities shown in black and beam shifts shown in red. The horizontal arrows indicating how each curve would shift for effective charge patch areas ranging from the minimum to maximum values of the effective capacitor plate area. The vertical arrows show estimated uncertainties in extracting shift values.

Figure 8 compares our experimental values of guiding probabilities and bypass beam deflections versus voltages obtained via this method with SIMION predictions. The beam current, amount of time the plates were allowed to discharge before injecting the beam, and effective patch dimensions, length × width in mm, that yield the best visual agreement are listed for each experimental dataset. The horizontal arrows for the 2° rotation data show how each curve would shift for effective patch areas ranging between the minimum to maximum values quoted above while the vertical arrows show uncertainties in determining the shifts.

Referring first to the left figure which shows the experimental data from Fig. 3 plus one dataset from Fig. 4, we note the following. (1) The 25 and 0.86 pA data and the SIMION voltage predictions are in reasonable agreement with regard to the onset and maximum guiding locations if it is assumed that the deposited charge is distributed over a 15 × 25 mm area, i.e., the initial charge redistributes in the transverse direction to the entire plate width and in the longitudinal direction to 50% longer than the geometric length. (2) However, in the same region for the 20 pA data to agree, the open magenta stars imply that the charge redistribution is less in both the transverse and the longitudinal directions. (3) At higher voltages, the shifted 20 pA curve shown by the open orange stars and dashed line shows agreement between the 20 and 25 pA curves. This implies that at later times there has been a further redistribution of charge in the transverse direction. We note that it is the areas, not the dimensions shown in the figure, that yield agreement and that our choice of charge being primarily redistributed in the transverse direction is in agreement with the electric fields shown in Fig. 2. Also, we point out that our SIMION model uses a static picture of the geometric charge distribution and does not account for any downstream charge deposition as the voltage slowly increases. Thus, the model shows a sharp onset in guiding whereas the data show a gradual increase. Finally, the decrease seen at the higher voltage end of the red curves is when a portion of the beam begins to impact the upper plate in Fig. 1. No guiding potential has been applied to account for this; therefore a “decrease in guiding” results.

The righthand figure shows our imaging data and SIMION predictions for the guiding probability and bypass beam deflection, the black and red curves and symbols respectively. The horizontal arrows show how the data would shift to the right or left for charge distributions ranging from the minimum to maximum areas previously discussed. Vertical arrows show estimated uncertainties in extracting the small beam deflections. For a charge distribution area given by the effective geometric patch length times roughly twice the effective width, the agreement between the measured and predicted guiding probabilities is similar to that seen in the left figure and reasonable for the bypass beam deflections. We note that for these data the beam current had to be estimated and is subject to a large uncertainty. This influences the magnitude of the guiding probability and the deposited charge which is used to determine the patch dimensions.

## Discussion

Three types of experimental studies and results from SIMION modeling for ion beam transport between two parallel glass plates have been described. The major findings from the high current study are that the amount of beam that impacts and charges the glass plate is time dependent, e.g., it decreases when the guiding probability is large, and that after the maximum beam transmission is reached and the plate voltage increases to more than half of the beam energy, the combined potentials of patches located on opposite plates cause a decrease in the transmitted beam well before the sharp decrease associated with beam blocking takes place. The lower current study was used to extract quantitative information about the charge decay times and the relative probabilities for the surface and bulk decay modes. The major finding here was that the two modes need to be treated separately, rather than averaged together, with respect to determining any residual charge. The final experimental study used a very low beam intensity to simultaneously image a guided beam and a bypass beam in order to correlate guiding probability with bypass beam deflection and obtain information about the amount of deposited charge and its distribution. To do this, the SIMION model was used to provide a snapshot of where and how much charge was deposited, how much beam was guided, and how much the bypass beam was deflected, all as a function of charge patch voltage. By combining this information with a simple equivalent electric circuit model consisting of capacitors, it was shown that quantitative agreement between the experimental data and SIMION results could be obtained by varying the area of the deposited charge distribution. The major findings from these combined data are that (a) the deposited charge tends to redistribute along the inner surface of the plate where it is deposited with migration through the bulk being far less important and (b) the redistribution is predominantly in the transverse, rather than longitudinal, direction which means that only a portion of the plate is charged.

This study provided new information from the particle transport through macroscopic object using the guiding phenomena. One improvement in any future studies would be to use a more intense guided beam and a larger angle of rotation in order to increase the patch voltage and therefore the deflections. Another would be to raster the bypass beam in order to sample the transverse charge distribution as a function of time. Also, rather than using a configuration where only the bottom edge of the glass plates was grounded, also grounding the top edges would give field symmetry and hence simplify any modeling. Additionally, reading the current from each plate independently would provide information about the initial and subsequent charge patch production.

## Data Availability

The datasets generated during and/or analyzed during the current study are available from the corresponding author on reasonable request.

## References

[1] Stolterfoht N, Bremer J-H, Hoffmann V, Hellhammer R, Fink D, Petrov A, Sulik B (2002). Transmission of 3 keV Ne 7+ ions through nanocapillaries etched in polymer foils: Evidence for capillary guiding. Phys. Rev. Lett..

[2] Lemell C, Burgdörfer J, Aumayr F (2013). Interaction of charged particles with insulating capillary targets: The guiding effect. Prog. Surf. Sci..

[3] Stolterfoht N, Yamazaki Y (2016). Guiding of charged particles through capillaries in insulating materials. Phys. Rep..

[4] Kojima TM (2018). Ion guiding in macro-size insulating capillaries: straight, tapered, and curved shapes. J. Phys. B.

[5] Stolterfoht N (2020). Simulations of ion-guiding through insulating nanocapillaries of varying diameter: Interpretation of experimental results. Atoms.

[6] Hellhammer R, Pesic ZD, Sobocinski P, Fink D, Bundesmann J, Stolterfoht N (2005). Guided transmission of highly charged ions through nanocapillaries in PET: Study of the energy dependence. Nucl. Instr. Methods Phys. Res. B..

[7] Vikor G, Rajendar Kumar RT, Pesic ZD, Stolterfoht N, Schuch R (2005). Guiding of slow highly charged ions by nanocapillaries in PET. Nucl. Instr. Method Phys. Res. B..

[8] Sahana MB, Skog P, Vikor G, Rajendar Kumar RT, Schuch R (2006). Guiding of highly charged ions by highly ordered Si O2 nanocapillaries. Phys. Rev. A..

[9] Matefi-Tempfli S, Matefi-Tempfli M, Piraux L, Juhasz Z, Biri S, Fekete E, Ivan I, Gall F, Sulik B, Vikor G, Palinkas J, Stolterfoht N (2005). Guided transmission of slow Ne6+ ions through the nanochannels of highly ordered anodic alumina. Nanotechnology.

[10] Skog P, Soroka IL, Johansson A, Schuch R (2007). Guiding of highly charged ions through Al_2_O_3_ nano-capillaries. Nucl. Instr. Method Phys. Res. B..

[11] Bereczky RJ, Kowarik G, Aumayr F, Tőkési K (2009). Transmission of 4.5 keV Ar9+ ions through a single glass macro-capillary. Nucl. Instr. Meth. Phys. Res. B.

[12] Kowarik RG, Bereczky RJ, Aumayr F, Tőkési K (2009). Production of a microbeam of slow highly charged ions with a single microscopic glass capillary. Nucl. Instr. Method Phys. Res. B.

[13] Gruber E, Kowarik G, Ladening F, Waclawek JP, Aumayr F, Bereczky RJ, Tőkési K, Gunacker P, Schweigler T, Lemell C, Burgdorfer J (2012). Temperature control of ion guiding through insulating capillaries. Phys. Rev. A.

[14] Giglio E, DuBois RD, Cassimi A, Tőkési K (2015). Low energy ion transmission through a conical insulating capillary with macroscopic dimensions. Nucl. Instr. Method Phys. Res. B.

[15] Rajta I, Nagy GUL, Bereczky RJ, Tőkési K (2015). Interaction of proton microbeam with the inner surface of a polytetrafluoroethylene macrocapillary. Nucl. Instr. Method Phys. Res. B..

[16] Nagy GUL, Rajta I, Bereczky RJ, Tőkési K (2015). Incident beam intensity dependence of the charge-up process of the guiding of 1 MeV proton microbeam through a Teflon microcapillary. Eur. Phys. J. D..

[17] Ikeda T, Yasuyuki K, Kojima TM, Iwai Y, Kambara T, Yamazaki Y, Hoshino M, Nebiki T, Narusawa T (2006). Production of a microbeam of slow highly charged ions with a tapered glass capillary. Appl. Phys. Lett..

[18] DuBois RD, Tőkési K, Giglio E (2019). Charge deposition, redistribution, and decay properties of insulating surfaces obtained from guiding of low-energy ions through capillaries. Phys. Rev. A.

[19] Giglio E, Tőkési K, DuBois RD (2019). Relaxation dynamics of charge patches formed inside an insulating capillary by ion impact. Nucl. Instr. Method Phys. Res. B.

[20] Giglio E, Guillous S, Cassimi A, Zhang HQ, Nagy GUL, Tőkési K (2017). Evolution of the electric potential of an insulator under charged particle impact. Phys. Rev. A.

[21] Giglio E, Guillous S, Cassimi A (2018). Ion-beam focusing by self-organized axis-symmetric potentials in insulating capillaries. Phys. Rev. A.

[22] Pokhil G, Petukhov V, Vokhmyanina KA (2006). Transportation and focusing of accelerated proton beams by means of dielectric channels. Izv. Akad. Nauk Ser. Fiz..

